# Differences Between STEMI and NSTEMI Complicated by Cardiogenic Shock: Insights From a Contemporary Multilevel Registry

**DOI:** 10.1016/j.jacadv.2026.103000

**Published:** 2026-07-17

**Authors:** Serdar Farhan, Sanjay Sivalokanathan, Abduljabar Adi, Sami Dalati, Daniel Miklin, Allan Lin, Jack Jnani, Niloufar Novin, Marcy Miller, Atul Bali, Emily Rodriguez, Emily Schultz, Sandrine Lebrun, Matthew Griffin, Matthew Pierce, Holger Thiele, Miguel Alvarez Villela

**Affiliations:** a Northwell Health, Cardiovascular Institute, New Hyde Park, New York, USA; b Lenox Hill Hospital at Northwell Health, New York, New York, USA; c Department of Cardiology, Mount Sinai Morningside, New York, New York, USA; d Zena and Michael A. Wiener Cardiovascular Institute, Icahn School of Medicine at Mount Sinai, New York, New York, USA; e North Shore University Hospital, Manhasset, New York, USA; f Heart Center Leipzig at Leipzig University, Department of Internal Medicine–Cardiology, Leipzig, Germany

**Keywords:** acute myocardial infarction, cardiac critical care, cardiogenic shock, coronary artery disease, mechanical circulatory support

## Abstract

**Background:**

An enhanced understanding is needed of the clinical trajectories seen in contemporary practice among patients with cardiogenic shock (CS) due to different acute myocardial infarction (AMI) types.

**Objectives:**

The objective of the study was to compare clinical characteristics, management strategies, and outcomes among patients with CS due to AMI with and without ST-segment elevation.

**Methods:**

All adults treated for CS due to STEMI or NSTEMI within a multilevel of care health system spanning 11 hospitals from 2016 to 2022 were included. The primary and secondary outcomes were 6-month and in-hospital all-cause mortality.

**Results:**

We identified 1,375 patients: 57% ST-segment elevation myocardial infarction with CS (STEMI-CS) and 43% non-STEMI-CS (NSTEMI-CS). STEMI-CS patients had more severe shock with more cardiac arrest and higher initial lactate whereas NSTEMI-CS patients were older with more comorbidities and more severe coronary disease. NSTEMI-CS patients received more coronary artery bypass grafting (33% vs 8%) and lower rates of percutaneous coronary intervention (35% vs 73%) in those undergoing left heart catheterization. Management patterns were consistent across hospital levels. Rates of in-hospital and 6-month mortality (STEMI-CS 37.1% vs NSTEMI-CS 34.0%; *P* = 0.53) were similar in both groups. On multivariable analysis, revascularization was associated with lower 6-month mortality in both groups, whereas intra-aortic balloon pump or percutaneous ventricular assist device use was associated with lower mortality only in STEMI-CS. Venoarterial extracorporeal membrane oxygenation was associated with increased mortality in both AMI types.

**Conclusions:**

STEMI and NSTEMI patients with CS have distinct clinical profiles and management strategies across all hospital levels. Although they experience similar rates of short and long-term mortality, associations between mechanical circulatory support use and outcomes differ by phenotype. These findings suggest that AMI type should inform management strategies and research design in CS.

Cardiogenic shock (CS) remains a severe complication of acute myocardial infarction (AMI), associated with poor survival despite significant advancements in coronary revascularization, mechanical circulatory support (MCS) devices, and intensive care unit management strategies.^1^, ^2^, ^3^, ^4^, ^5^, ^6^, ^7^

Based on electrocardiographic features, AMI is classified as either ST-segment elevation myocardial infarction (STEMI) or non-STEMI (NSTEMI). This classification reflects distinct underlying pathogenetic processes and serves to guide early risk stratification and management decisions.^8^ Although STEMI is usually associated with a complete coronary occlusion and is treated with emergent reperfusion, NSTEMI usually reflects a critical coronary stenosis with preserved vessel patency and has a less well-defined management strategy.^9^ Existing literature suggests that compared with STEMI patients, those with NSTEMI more commonly have multivessel coronary disease, older age, and more comorbidities. Hence, the development of CS in each entity is thought to be related to different physiological factors and occurs more commonly among patients with STEMI.^1^, ^2^, ^3^, ^4^, ^5^, ^6^, ^7^, ^8^, ^9^, ^10^

With the growth of the field of CS, further clinical trials and observational studies testing novel MCS devices and management strategies are likely to follow. Hence, a clearer understanding of phenotype-specific differences in the contemporary practice landscape is needed to refine treatment algorithms and improve patient selection for future studies. Clinical trials in AMI complicated by CS (AMI-CS) have predominantly or exclusively enrolled patients with STEMI with CS (STEMI-CS),^5^^,^^11^^,^^12^ and observational studies are limited by small overall sample sizes or heterogeneous data depth, resulting in an ongoing paucity of evidence for the management of NSTEMI with CS (NSTEMI-CS).^2^^,^^3^^,^^5^^,^^6^^,^^13^, ^14^, ^15^

In this study, we aim to provide a contemporary, comprehensive comparison of the clinical characteristics, management strategies, and outcomes of patients with STEMI-CS and NSTEMI-CS treated within a multilevel health system in North America.

## Methods

### Study design and population

This study utilized data from the Northwell-Shock registry, a multilevel registry of patients with CS treated at 11 hospitals within the metropolitan New York area, between January 2016 and August 2022.^16^ This cohort includes 6 hospitals with 24/7 percutaneous coronary intervention (PCI) capability, of which 4 have on-site cardiac surgery support and extracorporeal membrane oxygenation (ECMO) capability, and one has transplant capability.

All adult patients (age ≥18 years) presenting with AMI complicated by CS were included. CS was identified using the International Classification of Diseases (ICD)-10 code R57.0. Patients were classified as having STEMI or NSTEMI based on ICD-10 primary and secondary discharge diagnoses. This strategy has been previously validated against chart review and demonstrates high positive predictive value and excellent agreement for AMI subtype classification.^17^ A complete list of ICD-10 codes used for each condition is found in Supplemental Table 1. Codes indicating unspecified myocardial infarction, type 2 myocardial infarction, chronic ischemic heart disease, and historical myocardial infarction were excluded. To ensure ICD-coding accuracy, a manual chart review was conducted on (92.2% STEMI-CS and 87.5% NSTEMI-CS patients).

The study was approved by the Institutional Review Board at Donald and Barbara Zucker University at Hofstra/Northwell, which granted a waiver of informed consent due to the retrospective nature of the research.

### Study outcomes

The primary outcome was 6-month all-cause mortality. The secondary outcome was all-cause in-hospital mortality. Vital status at 6 months among patients who survived to discharge was ascertained by manual chart review of the Northwell Health electronic record; patients without a documented death and with at least 1 in-network encounter beyond 6 months were classified as alive. Those without documentation were classified as lost to follow-up. A STROBE (Strengthening the reporting of observational studies in epidemiology) diagram depicting the subjects included and their follow-up (Supplemental Figure 5), as well as a comparison of baseline characteristics between patients lost-to-follow-up and those with complete follow-up information (Supplemental Table 2) is provided in the Supplemental Appendix.

### Data collection and variables

Clinical characteristics, management procedures, and outcomes at hospital discharge were collected for all patients. For patients undergoing diagnostic left heart catheterization (LHC), all angiograms were reviewed by an interventional cardiologist involved with the study (M.A.V., S.L., E.S., and A.B. from the author list), who adjudicated the coronary anatomy, including culprit vessel, number of diseased vessels, and the presence of chronic total occlusions (CTOs). For those undergoing PCI, basic procedural details, including the target vessel and the use of intravascular ultrasound, were collected.

Vital signs, laboratory parameters, and hemodynamics were collected at the time of admission (initial) and at the point of the worst value for each specific parameter (worst). The vasoactive-inotropic score was calculated using the aggregate type and dosage of inotropes and vasopressors administered concomitantly, measured at admission and at the point of maximum dosing.^18^

The presence of pulmonary artery catheter (PAC) and different MCS devices was determined using ICD-10 procedural codes (Supplemental Table 1). Shock severity was defined at admission using the CS Working Group modification of the Society for Cardiovascular Angiography and Interventions (SCAI) SHOCK stages classification. The Acute Physiology and Chronic Health Evaluation version IV was calculated for all patients at the time of intensive care unit admission.^19^^,^^20^

### Statistical analysis

Continuous variables are reported as means ± SD or medians with IQR, and categorical variables as counts and percentages. Comparisons between groups (STEMI-CS vs NSTEMI-CS) were made using Student’s t-tests or Mann-Whitney U tests for continuous variables and chi-square or Fisher exact tests for categorical variables. Descriptive comparisons used available-case data, with the denominator for each variable using nonmissing observations. Multivariable logistic regression, stratified by AMI type, was performed to examine the association of both categorical and continuous variables with outcomes. Identical multivariable logistic regression models were fit separately for 6-month all-cause mortality (primary) and in-hospital mortality (secondary). Patients lost to follow-up at 6 months were excluded from the 6-month model only. Receipt of acute renal replacement therapy (RRT) during the index admission was included as a covariate in both models and was defined by ICD-10 codes 5A1D00Z, 5A1D60Z, 5A1D70Z, 5A1D80Z, and 5A1D90Z (continuous or intermittent dialysis, or ultrafiltration). Continuous variables were assessed as quartiles of their respective distribution to avoid imposing a linearity assumption, and all other covariates were treated as categorical. Missing covariate values were handled by multiple imputation by chained equations; outcomes were not imputed, and cases with a missing outcome were excluded from the relevant model to avoid introducing artificial outcome-covariate associations. A 2-sided *P* value of <0.05 was considered statistically significant. Statistical analyses were performed using GraphPad Prism (GraphPad Software; version 10.6.1) and R (R foundation for Statistical Computing; version 4.5.0).

Treatment-outcome associations are reported as adjusted odds ratios with 95% CIs and are framed as exploratory and hypothesis-generating, given the observational design.

## Results

We included 1,375 patients with AMI complicated by CS due to STEMI-CS (n = 790) and NSTEMI-CS (n = 585).

### Clinical profile and presentation

STEMI-CS patients were significantly younger (67.2 ± 13.0 vs 71.7 ± 12.0; *P* < 0.001), and males comprised the majority in both groups: 68.2% in STEMI (n = 539) and 69.1% in NSTEMI (n = 404); *P* = 0.742.

The NSTEMI cohort presented with a markedly higher burden of chronic comorbid conditions. The prevalence of chronic kidney disease, diabetes mellitus, prior coronary artery disease (CAD) (57.1% vs 35.9%; *P* < 0.001), hypertension, and atrial fibrillation (26.7% vs 19.2%; *P* = 0.001) were all higher in the NSTEMI group (Table 1). In addition, a significantly higher Charlson Comorbidity Index was seen among NSTEMI-CS patients compared with the STEMI-CS cohort (6.3 ± 3.2 vs 5.0 ± 3.0; *P* < 0.001). A greater proportion of NSTEMI-CS patients underwent interhospital transfer (47% vs 39%); however, only 1.3% of STEMI-CS patients were treated at non-PCI centers, likely because of prehospital triaging. Patients with STEMI-CS experienced higher rates of cardiac arrest at admission (6.6% vs 2.9%; *P* = 0.002) than those with NSTEMI-CS.

**Table 1:** Baseline Characteristics and Presenting Clinical Profile of Patients With STEMI-CS and NSTEMI-CS

	STEMI-CS (n = 790)	NSTEMI-CS (n = 585)	*P* Value
Age (years) hospital level	67.2 ± 13.0	71.7 ± 12.0	<0.001
Male	539 (68.2%)	404 (69.1%)	0.742
BMI (kg/m^2^)	26.6 (24.0, 30.2)	26.5 (23.1, 30.4)	0.220
Race			0.013
African American/Black	63 (8.0%)	67 (11.5%)	
Asian	67 (8.5%)	69 (11.8%)	
Other/multiracial	151 (19.1%)	94 (16.1%)	
White	443 (56.1%)	304 (52.0%)	
Transferred from another hospital	307 (38.9%)	276 (47.2%)	0.002
Hospital level			<0.001
Transplant center	311 (39.4%)	251 (42.9%)	0.187
ECMO center	425 (53.8%)	261 (44.6%)	0.001
IABP and pVAD center	44 (5.6%)	38 (6.5%)	0.473
No PCI center	10 (1.3%)	35 (6.0%)	<0.001
Comorbidities			
Hypertension	460 (58.2%)	413 (70.6%)	<0.001
Chronic kidney disease	102 (12.9%)	132 (22.6%)	<0.001
Coronary artery disease	284 (35.9%)	334 (57.1%)	<0.001
Type 2 diabetes mellitus	265 (33.5%)	294 (50.3%)	<0.001
Atrial fibrillation	152 (19.2%)	156 (26.7%)	0.001
CCI total score	5.0 ± 3.0	6.3 ± 3.2	<0.001
Heart rate on admission (beats/min)	82.0 (69.0, 97.0)	82.0 (68.0, 97.0)	0.766
Systolic blood pressure on admission (mm Hg)	98.0 (81.0, 118.0)	100.0 (85.0, 116.0)	0.282
Mean arterial pressure on admission (mm Hg)	69.3 (58.4, 82.0)	70.3 (60.3, 82.0)	0.497
LVEF, initial (%)	30.0 (20.0, 40.0)	30.0 (20.0, 40.0)	0.840
APACHE score, initial	85.3 ± 39.7	83.7 ± 34.4	0.522
Cardiac arrest on admission	52 (6.6%)	17 (2.9%)	0.002
SCAI stage on admission			<0.001
A	50 (6.3%)	153 (26.2%)	<0.001
B	108 (13.7%)	103 (17.6%)	0.040
C	117 (14.8%)	72 (12.3%)	0.196
D	231 (29.2%)	117 (20.0%)	<0.001
E	263 (33.3%)	121 (20.7%)	<0.001

Values are mean ± SD, median (Q1, Q3), or n (%). *P* values reflect Student's t-test, Mann-Whitney U test, or chi-square/Fisher exact test, as appropriate.

APACHE = Acute Physiology and Chronic Health Evaluation score; BMI = Body Mass Index; CCI = Charlson Comorbidity Index; ECMO = extracorporeal membrane oxygenation; IABP = intra-aortic balloon pump; LVEF = left ventricle ejection fraction; NSTEMI-CS = non–ST-segment elevation myocardial infarction with cardiogenic shock; PCI = percutaneous coronary intervention; pVAD = percutaneous ventricular assist device; SCAI = Society for Cardiovascular Angiography and Interventions; STEMI-CS = ST-segment elevation myocardial infarction with cardiogenic shock.

**Figure 1:**
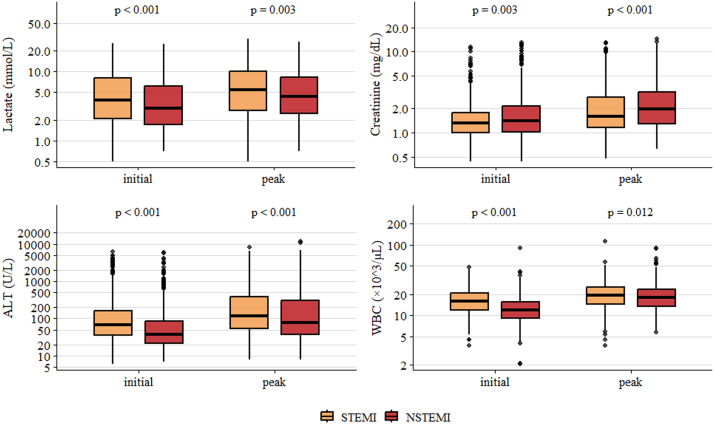
Initial and Peak Laboratory Values Tukey Box plots comparing admission and peak laboratory values in patients with ST-segment elevation myocardial infarction (STEMI) vs non–ST-segment elevation myocardial infarction (NSTEMI). Panels display lactate, creatinine, alanine aminotransferase (ALT), and white blood cell count (WBC), shown on logarithmic scales. *P* values reflect Mann-Whitney U test.

There was a notable difference in presenting shock severity as measured by the SCAI staging system (Table 1, Supplemental Figure 3), where advanced stages (D/E) were more prevalent in STEMI-CS patients (62.5% vs 40.7%; *P* < 0.001), and early shock with SCAI stage A or B (*P* = 0.040 for B), was more common in NSTEMI-CS. Initial heart rate and arterial blood pressure as well as invasive hemodynamic parameters, including pulmonary artery pressures, central venous pressure, and cardiac index, were all comparable between both groups. Despite this, patients with STEMI-CS exhibited significantly greater metabolic disturbance (Figure 1), evidenced by higher lactate levels at admission (3.9 mmol/L [2.1-8.1] vs 3.0 mmol/L [1.7-6.1]; *P* < 0.001) and peak level (5.4 mmol/L [2.7-10.1] vs 4.4 mmol/L [2.5-8.2]; *P* = 0.003) as well as higher admission and peak alanine aminotransferase levels (69.0 U/L [35.0-161.0] vs 37.0 U/L [22.0-84.0]; *P* < 0.001) and (116.0 U/L [54.0-377.5] vs 78.0 U/L [37.0-312.0]; *P* < 0.001), respectively. On the other hand, renal dysfunction was more pronounced in NSTEMI-CS patients, as demonstrated by higher admission (1.4 mg/dL (1.0-2.1) vs 1.3 (1.0-1.8); *P* = 0.003) and peak creatinine (1.9 mg/dL [1.3-3.2] vs 1.6 mg/dL [1.1-2.7]; *P* < 0.001) levels. In addition, NSTEMI-CS patients demonstrated lower initial (11.5 g/dL (9.7-13.4) vs 12.6 g/dL (10.5-14.0); *P* < 0.001) and nadir (8.3 g/dL (7.4-9.8) vs 9.3 g/dL (7.6-11.7); *P* < 0.001) hemoglobin levels.

### Coronary anatomy and revascularization patterns

Patients with STEMI-CS underwent diagnostic LHC more frequently (87.0% vs 77.9%, *P* < 0.001). However, NSTEMI-CS patients who underwent LHC had a substantially higher burden of CAD, with a greater average number of diseased vessels (2.5 ± 1.1 vs 1.9 ± 1.0; *P* < 0.001), a higher prevalence of multivessel CAD (>1 diseased vessel) (82.5% vs 55.6%; *P* < 0.001), and more frequent involvement of the left main coronary artery compared with STEMI-CS patients (30.7% vs 12.2%; *P* < 0.001).

Concurrent CTO were more common in NSTEMI-CS, present in 33.3% of patients compared to 9.6% of STEMI-CS patients (*P* < 0.001). NSTEMI-CS had higher CTO prevalence across all coronary vessels, with the right coronary artery most frequently affected (22.1% vs 5.8%), followed by the left anterior descending (13.8% vs 3.3%) and left circumflex arteries (9.6% vs 2.5%) (*P* < 0.001 for all).

In contrast, acute coronary occlusion was significantly more frequent in STEMI-CS patients (67.5% vs 18.2%; *P* < 0.001) (Table 2, Supplemental Figure 4), and the most involved vessels were the left anterior descending artery (STEMI-CS 38.7% vs NSTEMI-CS 6.1%). Of those who received LHC, PCI was performed in 72.8% of STEMI-CS patients, compared to only 35.1% of NSTEMI-CS patients (*P* < 0.001). Meanwhile, coronary artery bypass grafting (CABG) was performed in 32.8% of NSTEMI-CS patients compared to 7.8% in STEMI-CS (Central Illustration). Among patients receiving PCI, NSTEMI-CS had significantly longer delays (1.0 [0.0-3.0] vs 0.0 [0.0-0.1] days; *P* = 0.010), but the average number of vessels treated during PCI (1.0 [1.0-1.0] vs 1.0 [1.0-1.0]; *P* = 0.070), as well as the rates of multivessel PCI were similar between groups (13.6% vs 19.4%, *P* = 0.071). PCI of the left main, on the other hand, was more frequent in NSTEMI-CS patients (17.5% vs 5.6%, *P* < 0.001). Intravascular ultrasound guidance use was low and similar between groups, approximately 11%.

**Table 2:** Coronary Angiography and Revascularization Characteristics

	STEMI-CS (N = 790)	NSTEMI-CS (N = 585)	*P* Value
CABG	62 (7.8%)	192 (32.8%)	<0.001
LHC: angiographic characteristics^a^	687 (87.0%)	456 (77.9%)	<0.001
Acute coronary occlusion	464 (67.5%)	83 (18.2%)	<0.001
Chronic total occlusion	66 (9.6%)	152 (33.3%)	<0.001
Number of diseased coronary vessels	1.9 ± 1.0	2.5 ± 1.1	<0.001
Multivessel CAD (>1 vessel)	382 (55.6%)	376 (82.5%)	<0.001
Left main involvement	84 (12.2%)	140 (30.7%)	<0.001
IVUS	81 (11.8%)	51 (11.2%)	0.848
PCI: angiographic characteristics^b^	500 (72.8%)	160 (35.1%)	<0.001
Days to PCI	0.0 (0.0, 0.1)	1.0 (0.0, 3.0)	0.010
Left main	28 (5.6%)	28 (17.5%)	<0.001
Left anterior descending	289 (57.8%)	73 (45.6%)	0.008
Left circumflex	95 (19.0%)	49 (30.6%)	0.002
Right coronary artery	156 (31.2%)	34 (21.2%)	0.017
Ramus	7 (1.4%)	6 (3.8%)	0.094
Bypass graft	2 (0.4%)	5 (3.1%)	0.011
Number of vessels treated with PCI	1.0 (1.0, 1.0)	1.0 (1.0, 1.0)	0.070
Multivessel PCI	68 (13.6%)	31 (19.4%)	0.071

Values are mean ± SD, median (Q1, Q3), or n (%). *P* values reflect Student's t-test, Mann-Whitney U test, or chi-square/Fisher exact test, as appropriate.

CABG = coronary artery bypass grafting; CAD = coronary artery disease; IVUS = intravascular ultrasound; LHC = left heart catheterization; other abbreviations as in Table 1.

a Percentages under "LHC: angiographic characteristics" use the number of patients who underwent LHC in each group as the denominator.

b Percentages under "PCI: angiographic characteristics" use the number of patients who underwent PCI in each group as the denominator. The PCI row itself also uses the number of patients who underwent LHC as the denominator (consistent with body text and Central Illustration). All other percentages use the full STEMI-CS or NSTEMI-CS cohort as the denominator.

**Central Illustration:**
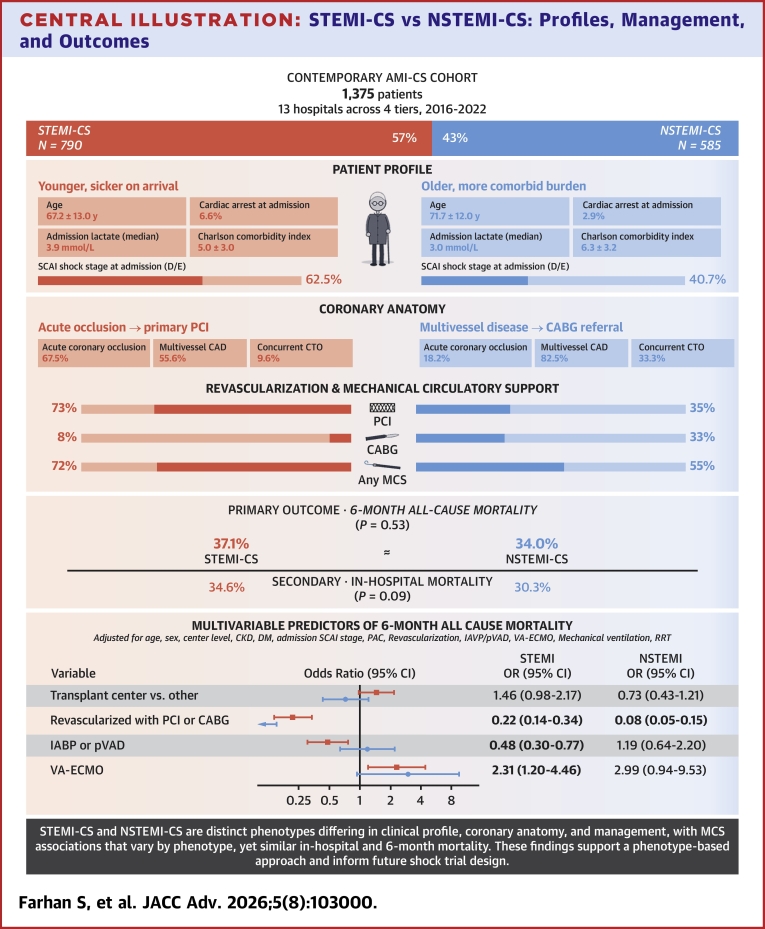
STEMI-CS vs NSTEMI-CS: Profiles, Management, and Outcomes STEMI-CS patients were younger with more frequent cardiac arrest and advanced shock; NSTEMI-CS patients were older with greater comorbidity, multivessel disease, and concurrent chronic total occlusion. PCI and MCS predominated in STEMI-CS, whereas CABG was more common in NSTEMI-CS. Six-month mortality was similar despite these distinct profiles (STEMI-CS 37.1% vs NSTEMI-CS 34.0%; *P* = 0.53). On multivariable analysis, revascularization was associated with lower mortality in both phenotypes. IABP or pVAD was associated with lower mortality in STEMI-CS only; VA-ECMO was associated with higher mortality in both groups. AMI-CS = acute myocardial infarction complicated by cardiogenic shock; CABG = coronary artery bypass grafting; CAD = coronary artery disease; CKD = chronic kidney disease; CTO = chronic total occlusion; DM = Diabetes mellitus; IABP = intra-aortic balloon pump; MCS = mechanical circulatory support; NSTEMI-CS = non–ST-segment elevation myocardial infarction with cardiogenic shock; PAC = pulmonary artery catheter; PCI = percutaneous coronary intervention; pVAD = percutaneous ventricular assist device; RRT = renal replacement therapy; SCAI = Society for Cardiovascular Angiography and Interventions; STEMI-CS = ST-segment elevation myocardial infarction with cardiogenic shock; VA-ECMO = venoarterial extracorporeal membrane oxygenation.

### Mechanical circulatory support and intensive care therapies

The majority of patients in both groups were treated at ECMO capable centers (93.2% STEMI and 87.5% NSTEMI); however, STEMI-CS patients were more likely to receive MCS (71.6% vs 54.5%; *P* < 0.001), including intra-aortic balloon pump (IABP) (54.4% vs 42.9%; *P* < 0.001), percutaneous ventricular assist device (pVAD) (23.4% vs 17.9%; *P* = 0.014), and venoarterial ECMO (8.7% vs 4.6%; *P* = 0.003) as well as combination of MCS devices during the hospital stay (Table 3). The time from admission to MCS initiation was also shorter among STEMI-CS patients (0.0 [0.0-0.0] vs 1.0 [0.0-2.0] days; *P* < 0.001).

**Table 3:** Mechanical Circulatory Support, Intensive Care Therapies, and Hospital Outcomes

	STEMI-CS (n = 790)	NSTEMI-CS (n = 585)	*P* Value
MCS	566 (71.6%)	319 (54.5%)	<0.001
Days to MCS initiation	0.0 (0.0, 0.0)	1.0 (0.0, 2.0)	<0.001
IABP	430 (54.4%)	251 (42.9%)	<0.001
pVAD	185 (23.4%)	105 (17.9%)	0.014
VA-ECMO	69 (8.7%)	27 (4.6%)	0.003
Received >1 MCS device	105 (13.3%)	56 (9.6%)	0.034
Inotropes received during hospitalization			
Dobutamine	203 (25.7%)	168 (28.7%)	0.212
Milrinone	117 (14.8%)	102 (17.4%)	0.188
Dopamine	50 (6.3%)	16 (2.7%)	0.002
Vasopressors received during hospitalization			
Norepinephrine	322 (40.8%)	231 (39.5%)	0.634
Epinephrine	102 (12.9%)	82 (14.0%)	0.552
Vasopressin	205 (25.9%)	172 (29.4%)	0.156
PAC	470 (59.5%)	337 (57.6%)	0.482
Days to PAC	0.0 (0.0, 0.5)	1.5 (0.0, 4.0)	<0.001
Mechanical ventilation	460 (58.2%)	257 (43.9%)	<0.001
Blood transfusion	263 (33.3%)	272 (46.5%)	<0.001
Renal replacement therapy	103 (13.0%)	96 (16.4%)	0.079
In-hospital mortality	273 (34.6%)	177 (30.3%)	0.093^a^
Mortality: all hospital levels	273 (34.6%)	177 (30.3%)	0.420^b^
Mortality: transplant hospital	120 (38.6%)	73 (29.1%)	0.093^b^
Mortality: ECMO hospital	135 (31.8%)	75 (28.7%)	>0.99^b^
Mortality: PCI hospital	11 (25%)	14 (36.8%)	>0.99^b^
Deceased by 6-months postdischarge (cumulative)	293 (37.1%)	199 (34.0%)	0.533
Discharge to skilled nursing facility, rehabilitation, or home health	285 (36.1%)	293 (50.1%)	<0.001
Length of stay (days)	7.0 (3.0, 15.0)	10.0 (5.0, 18.0)	<0.001

Values are mean ± SD, median (Q1, Q3), or n (%). *P*-values reflect Student's t-test, Mann-Whitney U test, or chi-square/Fisher exact test, as appropriate.

MCS = mechanical circulatory support; PAC = pulmonary artery catheter; VA-ECMO = venoarterial extracorporeal membrane oxygenation; other abbreviations as in Table 1.

a Uncorrected *P* value for the unstratified comparison.

b Bonferroni-corrected for 4 within-center comparisons (STEMI-CS vs NSTEMI-CS); percentages are calculated within each hospital level.

Hemodynamic monitoring with PAC was similar between groups, but these devices were used earlier among STEMI-CS patients (0.0 [0.0-0.5] vs 1.5 [0.0-4.0] days; *P* < 0.001). Vasoactive therapy, including overall intensity as measured by vasoactive inotropic score, was comparable between groups, although dopamine use was higher in STEMI-CS (6.3% vs 2.7%; *P* = 0.002).

Mechanical ventilation was more frequently required in STEMI-CS (58.2% vs 43.9%; *P* < 0.001). In contrast, hospital length of stay was longer among NSTEMI-CS patients (10.0 [5.0-18.0] vs 7.0 [3.0-15.0] days; *P* < 0.001).

### Management patterns across hospital levels

**Figure 2:**
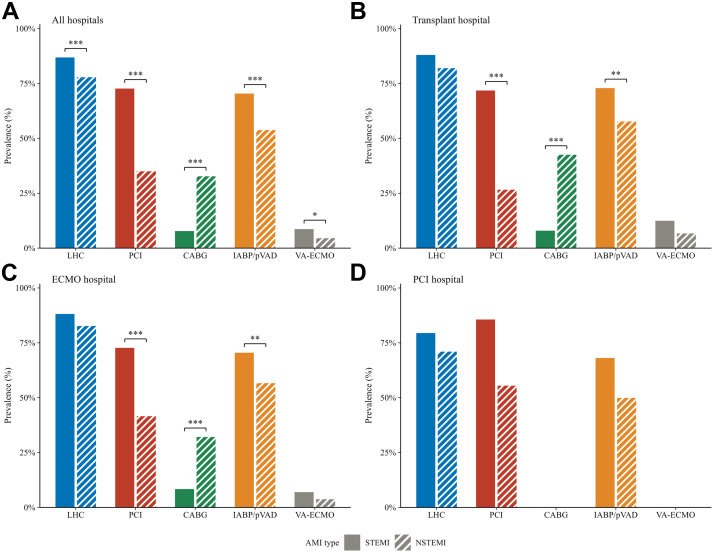
Procedural Use by Hospital Capability Level and Acute Myocardial Infarction Complicated by Cardiogenic Shock Subtype Proportion of patients with acute myocardial infarction complicated by cardiogenic shock (AMI-CS) undergoing each procedure, stratified by AMI-CS subtype and receiving-hospital level: (A; STEMI-CS n = 780, NSTEMI-CS n = 550) all centers capable of transplant/extracorporeal membrane oxygenation (ECMO)/percutaneous coronary intervention (PCI), (B; STEMI-CS n = 311, NSTEMI-CS n = 251) transplant-capable centers, (C; STEMI-CS n = 425, NSTEMI-CS n = 261) ECMO-capable centers, and (D; STEMI-CS n = 44, NSTEMI-CS n = 38) PCI-only centers. Bars are color-coded by procedure; solid fills denote STEMI-CS and hatched fills denote NSTEMI-CS. STEMI-CS vs NSTEMI-CS prevalence was compared within each level using the chi-square or Fisher exact test, with Bonferroni correction for the 5 procedures tested per level. ∗*P* < 0.05, ∗∗*P* < 0.01, ∗∗∗*P* < 0.001. AMI = acute myocardial infarction; CABG = coronary artery bypass grafting; IABP/pVAD = intra-aortic balloon pump or percutaneous ventricular assist device; LHC = left heart catheterization; NSTEMI = non–ST-elevation myocardial infarction; PCI = percutaneous coronary intervention; STEMI = ST-elevation myocardial infarction; VA-ECMO = venoarterial ECMO.

Patterns of management at each hospital level consistently differed between STEMI-CS and NSTEMI-CS (Figure 2). At both transplant- and ECMO-capable centers, STEMI-CS patients underwent PCI more often than NSTEMI-CS patients (transplant: 71.9% vs 26.7%; ECMO: 72.8% vs 41.7%; both *P* < 0.001) and received IABP or pVAD more often (transplant: 73.0% vs 57.8%; ECMO: 70.6% vs 56.7%; both *P* ≤ 0.001). NSTEMI-CS patients, by contrast, were preferentially treated with CABG (transplant: 42.6% vs 8.0%; ECMO: 32.2% vs 8.5%; *P* for both <0.001). At PCI-capable centers without on-site ECMO or cardiac surgery, the same patterns were observed for PCI and MCS but did not reach significance. PAC use was uniformly lower at PCI-only centers than at higher-level centers in both phenotypes (STEMI-CS 22.7% vs 70.7% at transplant; NSTEMI-CS 31.6% vs 60.2%; *P* for both <0.001).

### Outcomes

**Figure 3:**
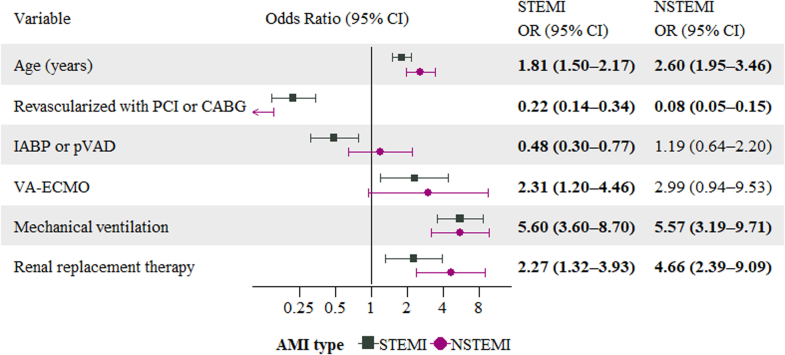
Multivariable Predictors of 6-Month Mortality by Acute Myocardial Infarction Complicated by Cardiogenic Shock Phenotype Multivariable logistic regression for cumulative all-cause mortality at 6 months from the index admission fit separately in STEMI-CS and NSTEMI-CS. Twelve covariates were modeled simultaneously. Portrayed are the significant associations (all 12 covariates are portrayed in Supplemental Figure 2); missing data were handled by multiple imputation by chained equations (MICE). Bold OR (95% CI) and *P* value entries indicate *P* < 0.05. The reference line is at OR = 1; the x-axis is log-scaled. Abbreviations as in Figure 2.

The primary outcome of 6-month all-cause mortality was similar between STEMI-CS and NSTEMI-CS (37.1% vs 34.0%; *P* = 0.53) (Table 3, Central Illustration). In the multivariable regression analysis (Figure 3, Supplemental Figure 1, Central Illustration), revascularization with PCI or CABG was the factor most strongly associated with decreased mortality in both groups: NSTEMI-CS (OR: 0.08 [0.05-0.15]), STEMI-CS (OR: 0.22 [0.14-0.34]; both *P* < 0.001). IABP or pVAD was associated with lower mortality in STEMI-CS only (OR: 0.48 [0.30-0.77]; *P* < 0.01), with no significant association in NSTEMI-CS (OR: 1.19 [0.64-2.20]). Meanwhile, ECMO was associated with higher mortality in STEMI-CS (OR: 2.31 [1.20-4.46]; *P* = 0.01) but not NSTEMI-CS (OR: 2.99 [0.94-9.53]; *P* = 0.06).

Older age, mechanical ventilation, and RRT were all associated with increased mortality in both groups.

In-hospital mortality, the secondary outcome, was also similar between both phenotypes (34.6% vs 30.3%; *P* = 0.09) (Table 3). In-hospital mortality did not differ between STEMI-CS and NSTEMI-CS across hospital levels (*P* = 0.353).

On multivariable analysis, the factors associated with this outcome were similar to those seen on the 6-month mortality model (Supplemental Figure 2), where revascularization with PCI or CABG was the factor most strongly associated with decreased mortality: NSTEMI-CS (OR: 0.09 [0.05-0.16]), STEMI-CS (OR: 0.24 [0.16-0.36]; both *P* < 0.001). IABP or pVAD was again predicted to be protective in STEMI-CS only (OR: 0.53 [0.34-0.83]; *P* < 0.01), with no association in NSTEMI-CS (OR: 1.19 [0.68-2.09]). Older age, mechanical ventilation, ECMO, and RRT were all associated with higher mortality in both groups (Supplemental Figures 1 and 2). Notably, management at a transplant capable hospital was associated with higher mortality in STEMI-CS (OR: 1.60 [1.09-2.32]; *P* = 0.02) but not NSTEMI-CS. Although SCAI stage C/D/E at admission was not associated with 6-month mortality, it seemed to be associated with lower mortality in NSTEMI-CS (OR: 0.58 [0.36-0.96]; *P* = 0.03).

## Discussion

This study provides a comprehensive characterization of patients with AMI-CS in a contemporary registry in North America. Our study is unique given the combination of large sample size and the inclusion of patient treated at hospitals with different resource levels.

Our findings highlight important pathophysiological differences between STEMI-CS and NSTEMI-CS that significantly enhance the current literature by showing how patients get treated at different levels of care and complement detailed clinical information with coronary anatomy and revascularization strategies.^21^, ^22^, ^23^, ^24^, ^25^

In this study, NSTEMI-CS patients carried a higher burden of chronic comorbidities and were older in age, whereas STEMI-CS patients were on average younger, but exhibited greater severity of shock on presentation with higher initial SCAI shock stages, higher lactate and alanine aminotransferase levels, as well as higher incidence of cardiac arrest. Hence, NSTEMI-CS appears to represent an ischemia-driven decompensation in chronically ill individuals, whereas STEMI-CS represents a sudden, catastrophic ischemic event in patients with fewer pre-existing conditions.

Along with this clinical profile, coronary angiographic findings differed significantly between the 2 groups. STEMI-CS patients had high rates of acute coronary occlusion, whereas NSTEMI-CS patients had higher rates of multivessel disease, left main involvement, and concurrent CTOs. Similar findings have been previously reported.^1^^,^^2^

These contrasting phenotypes corresponded with distinct management patterns that remained consistent across hospitals with different resource levels. STEMI-CS patients more frequently underwent diagnostic LHC and PCI. They were also treated earlier and more aggressively with all MCS device types and received earlier placement of PACs. NSTEMI-CS patients on the other hand, had longer delays to LHC and were preferentially treated with CABG. The delay observed in invasive coronary work-up may be related to their lower presenting shock severity as well as a preference among clinicians for initial stabilization before revascularization in this group. The higher rates of referral to CABG on the other hand are consistent with the higher burden of complex multivessel disease among NSTEMI-CS patients.^23^

Despite these delays, revascularization remained strongly associated with decreased in-hospital and 6-month mortality in both NSTEMI-CS and STEMI-CS, indicating that this should be a paramount target in the management of AMI-CS.

The use of MCS in AMI-related CS has increased substantially over time despite limited evidence supporting its benefit.^26^^,^^27^ To date, randomized trials have largely demonstrated neutral results, with the notable exception of the DanGer-SHOCK trial, which enrolled a highly selected population of patients with STEMI-CS.^11^^,^^13^ More recently, a patient-level meta-analysis of randomized trials evaluating ECMO and pVAD suggested that any potential benefit of MCS may be confined to patients with STEMI-CS, particularly in the absence of hypoxic brain injury.^11^ Consistent with this evidence, we observed a phenotype-specific association between the use of MCS and mortality, where IABP or pVAD use was associated with lower mortality in STEMI-CS but not in NSTEMI-CS. This differential effect could be influenced by the earlier initiation of MCS in STEMI-CS patients, but also likely reflects the underlying pathophysiologic differences between the phenotypes. Venoarterial ECMO on the other hand showed either no significant association or an association with higher mortality in both phenotypes. Although this may be the result of residual bias in our adjusted model, as sicker patients receive ECMO treatment more often, this pattern is also consistent with prior results seen in the ECLS-SHOCK and ECMO-CS trials.^15^

Few observational studies in AMI-CS have reported outcomes beyond initial hospitalization or 30 days. In this study, we report on alive status at 6 months among survivors of index hospitalization. Despite the observed differences, mortality rates at both time points were similar between STEMI-CS and NSTEMI-CS. Most of the deaths were concentrated during the initial hospital stay and outcomes did not differ substantially for patients treated at different levels of care. In fact, treatment at the transplant-capable center was associated with higher mortality in STEMI-CS. This phenomenon has been previously described and may reflect the systematic transfer of sicker patients toward the higher levels of care for advanced management.^28^

Our study reflects the modern era of CS management with interhospital transfers, dedicated shock teams, and widespread use of MCS. Our results, therefore, provide important insight into how current management strategies interact with infarct phenotype.

Overall, our findings reinforce the perspective that STEMI-CS and NSTEMI-CS should be regarded as biologically and clinically distinct entities rather than mere variants of a single condition. Consequently, future clinical trials should not perceive AMI-CS as a homogeneous population, and should aim to stratify patients by AMI type. Failure to account for these distinct pathophysiologic substrates may partly explain the neutral results observed in prior CS trials.

### Study Limitations

Our study should be interpreted in light of the following limitations. Our registry is based on retrospective observational data and is subject to the biases inherent to this study design. Furthermore, despite the multicenter design, the data are derived from a single health system (Northwell Health), thereby limiting generalizability to other health systems. Finally, we applied ICD-10 codes for diagnosis of CS, STEMI, and NSTEMI as well as other clinical variables which might lead to inaccuracies in patients’ classification. The associations observed between revascularization, MCS use, and improved outcomes must be interpreted with caution. Patients receiving these interventions had to survive long enough in hospital to receive them, raising the possibility of immortal-time bias. In addition, treatment selection in CS is influenced by patient-level factors that may not be fully captured by recorded covariates (confounding by indication and unmeasured confounding). A fully time-aware causal framework is beyond the scope of this descriptive cohort analysis. Treatment-outcome associations should therefore be regarded as exploratory and hypothesis-generating. Six-month vital status was ascertained by manual chart review of the Northwell Health electronic record; out-of-network deaths may therefore be under ascertained. Patients lost to follow-up were excluded from the 6-month multivariable model. Both approaches potentially bias the 6-month mortality estimate toward the null value.

## Conclusions

In this contemporary multilevel of care cohort of patients with AMI complicated by CS, STEMI-CS and NSTEMI-CS represent distinct clinical phenotypes with important differences in patient characteristics, coronary anatomy, management strategies, and response to therapy. Despite these differences, in-hospital and 6-month mortality were similar between phenotypes. These findings support a phenotype-based approach to the management of AMI-related CS and should inform the design of future clinical trials.

Perspectives **COMPETENCY IN MEDICAL KNOWLEDGE:** STEMI and NSTEMI complicated by CS represent 2 distinct clinical phenotypes despite sharing a diagnostic label of AMI-CS. Patients with STEMI-CS tend to be younger and present with more severe hemodynamic instability and higher incidence of cardiac arrest. In contrast, patients with NSTEMI-CS are generally older, have more comorbidities, and more frequently exhibit multivessel and left main CAD, often requiring surgical revascularization. Although revascularization is associated with decreased mortality in both phenotypes, the use of pVAD or IABP was associated with lower mortality only in STEMI-CS. These findings highlight the need for phenotype-specific protocols and tailored use of MCS.

**TRANSLATIONAL OUTLOOK:** Our study reveals distinct biological underpinnings for STEMI-CS and NSTEMI-CS, opening important translational research avenues. Future efforts should focus on differential mechanisms in these phenotypes, from inflammation to myocardial recovery. This foundational understanding can uncover specific diagnostic biomarkers and novel therapeutic targets. The distinct associations with MCS demand further study into how early hemodynamic support impacts reperfusion and systemic inflammation uniquely across these AMI-CS types. Ultimately, future clinical trials should consider stratifying subjects by AMI type, to isolate the true effects of different interventions in specific patient phenotypes.

## Funding support and author disclosures

The authors have reported that they have no relationships relevant to the contents of this paper to disclose.
