# Supplementary material for: Differences Between STEMI and NSTEMI Complicated by Cardiogenic Shock: Insights From a Contemporary Multilevel Registry
Source: JACC Adv. 2026 Jul 17;5(8):103000. doi: 10.1016/j.jacadv.2026.103000 (PMC13400863; doi:10.1016/j.jacadv.2026.103000)
Supplement: Supplemental Material [file mmc1.pdf]

# Supplementary Appendix

| Variable | STEMI OR (95% CI) | STEMI P | NSTEMI OR (95% CI) | NSTEMI P |
|---|---|---|---|---|
| Age (years) | **1.81 (1.50–2.17)** | **<0.001** | **2.60 (1.95–3.46)** | **<0.001** |
| Sex: Female | 1.30 (0.86–1.96) | 0.210 | 1.04 (0.61–1.77) | 0.890 |
| Transplant hospital vs. other | 1.46 (0.98–2.17) | 0.063 | 0.73 (0.43–1.21) | 0.221 |
| Chronic kidney disease | 0.62 (0.36–1.06) | 0.080 | 0.62 (0.35–1.12) | 0.114 |
| Diabetes mellitus | 1.32 (0.87–1.99) | 0.186 | 1.11 (0.67–1.85) | 0.677 |
| SCAI stage C/D/E on admission | 1.16 (0.68–1.98) | 0.579 | 0.65 (0.38–1.12) | 0.123 |
| PAC | 0.89 (0.57–1.38) | 0.600 | 0.58 (0.33–1.04) | 0.066 |
| Revascularized with PCI or CABG | **0.22 (0.14–0.34)** | **<0.001** | **0.08 (0.05–0.15)** | **<0.001** |
| IABP or pVAD | **0.48 (0.30–0.77)** | **0.002** | 1.19 (0.64–2.20) | 0.587 |
| VA-ECMO | **2.31 (1.20–4.46)** | **0.013** | 2.99 (0.94–9.53) | 0.064 |
| Mechanical ventilation | **5.60 (3.60–8.70)** | **<0.001** | **5.57 (3.19–9.71)** | **<0.001** |
| Renal replacement therapy | **2.27 (1.32–3.93)** | **0.003** | **4.66 (2.39–9.09)** | **<0.001** |

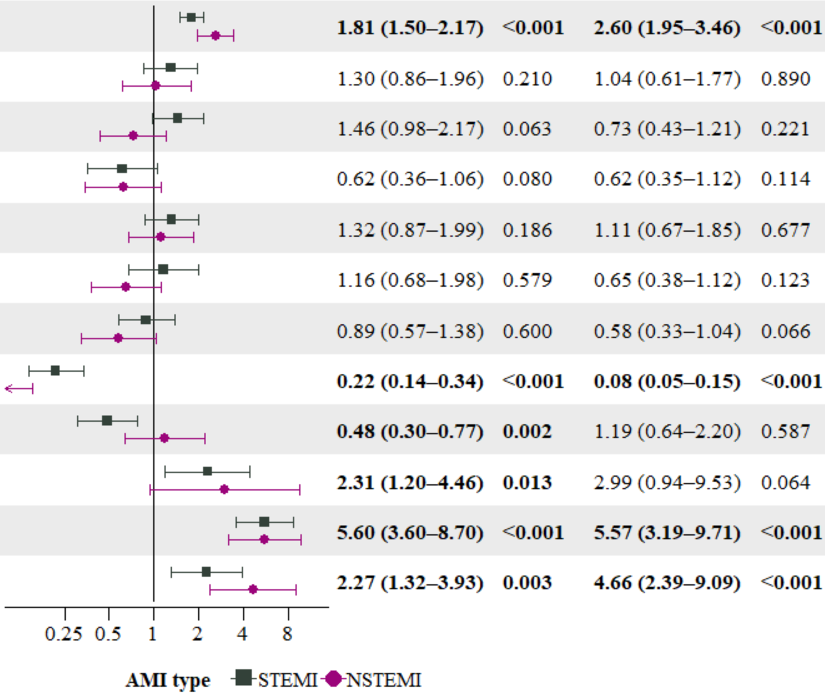


**Supplemental Figure 1. Multivariable predictors of 6-month mortality by AMI-CS phenotype**

Multivariable logistic regression model that adjusted for 12 candidate covariates. Models fit separately in STEMI-CS and NSTEMI-CS; missing data were handled by Multiple Imputation by Chained Equations (MICE). Bold OR (95% CI) entries indicate the subtype in which that covariate reached $p < 0.05$. The reference line is at OR=1; the x-axis is log-scaled.

AMI-CS = acute myocardial infarction complicated by cardiogenic shock; CABG = coronary artery bypass grafting; IABP = intra-aortic balloon pump; PCI = percutaneous coronary intervention; pVAD = percutaneous ventricular assist device; SCAI = Society for Cardiovascular

Angiography and Interventions; VA-ECMO = venoarterial extracorporeal membrane oxygenation.

| Variable | STEMI OR (95% CI) | STEMI P | NSTEMI OR (95% CI) | NSTEMI P |
|---|---|---|---|---|
| Age (years) | **1.64 (1.38–1.96)** | **<0.001** | **1.96 (1.52–2.54)** | **<0.001** |
| Sex: Female | 1.23 (0.84–1.82) | 0.288 | 1.18 (0.73–1.90) | 0.502 |
| Transplant hospital vs. other | **1.60 (1.09–2.32)** | **0.015** | 1.00 (0.63–1.61) | 0.988 |
| Chronic kidney disease | 0.72 (0.42–1.21) | 0.209 | 0.62 (0.36–1.06) | 0.081 |
| Diabetes mellitus | 0.94 (0.64–1.39) | 0.769 | 0.89 (0.57–1.41) | 0.621 |
| SCAI stage C/D/E on admission | 1.27 (0.76–2.12) | 0.364 | **0.58 (0.36–0.96)** | **0.034** |
| PAC | 0.92 (0.61–1.41) | 0.715 | 0.63 (0.37–1.07) | 0.084 |
| Revascularized with PCI or CABG | **0.24 (0.16–0.36)** | **<0.001** | **0.09 (0.05–0.16)** | **<0.001** |
| IABP or pVAD | **0.53 (0.34–0.83)** | **0.005** | 1.19 (0.68–2.09) | 0.541 |
| VA-ECMO | **2.03 (1.10–3.75)** | **0.023** | **2.93 (1.01–8.52)** | **0.049** |
| Mechanical ventilation | **6.53 (4.22–10.11)** | **<0.001** | **4.75 (2.88–7.86)** | **<0.001** |
| Renal replacement therapy | **2.50 (1.50–4.16)** | **<0.001** | **3.92 (2.16–7.14)** | **<0.001** |

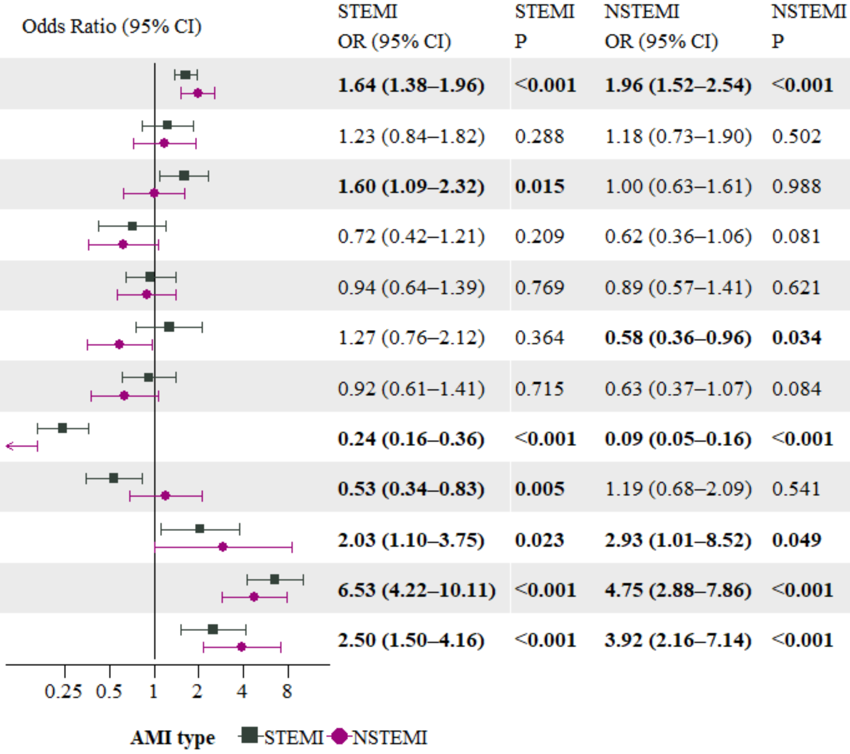


**Supplemental Figure 2. Multivariable predictors of in-hospital mortality by AMI-CS phenotype**

Multivariable logistic regression model that adjusted for 12 candidate covariates. Models fit separately in STEMI-CS and NSTEMI-CS; missing data were handled by Multiple Imputation by Chained Equations (MICE). Bold OR (95% CI) entries indicate the subtype in which that covariate reached $p < 0.05$. The reference line is at OR=1; the x-axis is log-scaled.

AMI-CS = acute myocardial infarction complicated by cardiogenic shock; CABG = coronary artery bypass grafting; IABP = intra-aortic balloon pump; PCI = percutaneous coronary intervention; SCAI = Society for Cardiovascular Angiography and Interventions; VA-ECMO = venoarterial extracorporeal membrane oxygenation.

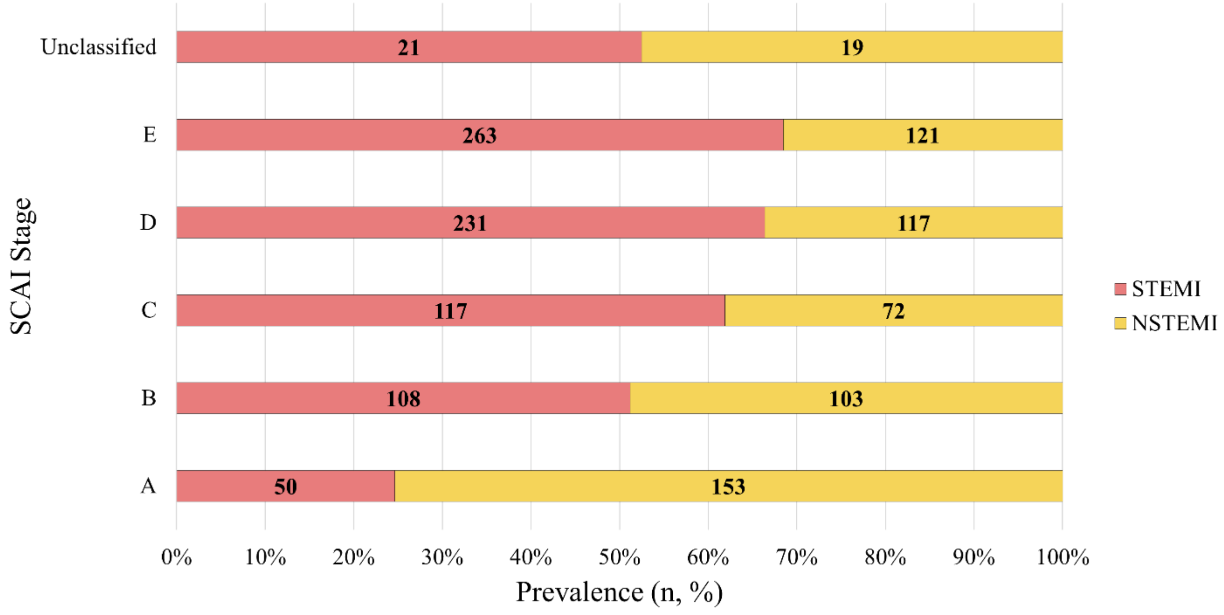


**Supplemental Figure 3. Initial SCAI stage**

Horizontal stacked bar chart comparing the distribution of Society for Cardiovascular Angiography and Interventions (SCAI) stages on admission in patients with ST-segment elevation myocardial infarction (STEMI) versus non–ST-segment elevation myocardial infarction (NSTEMI), with absolute counts ‘n’ displayed within each category.

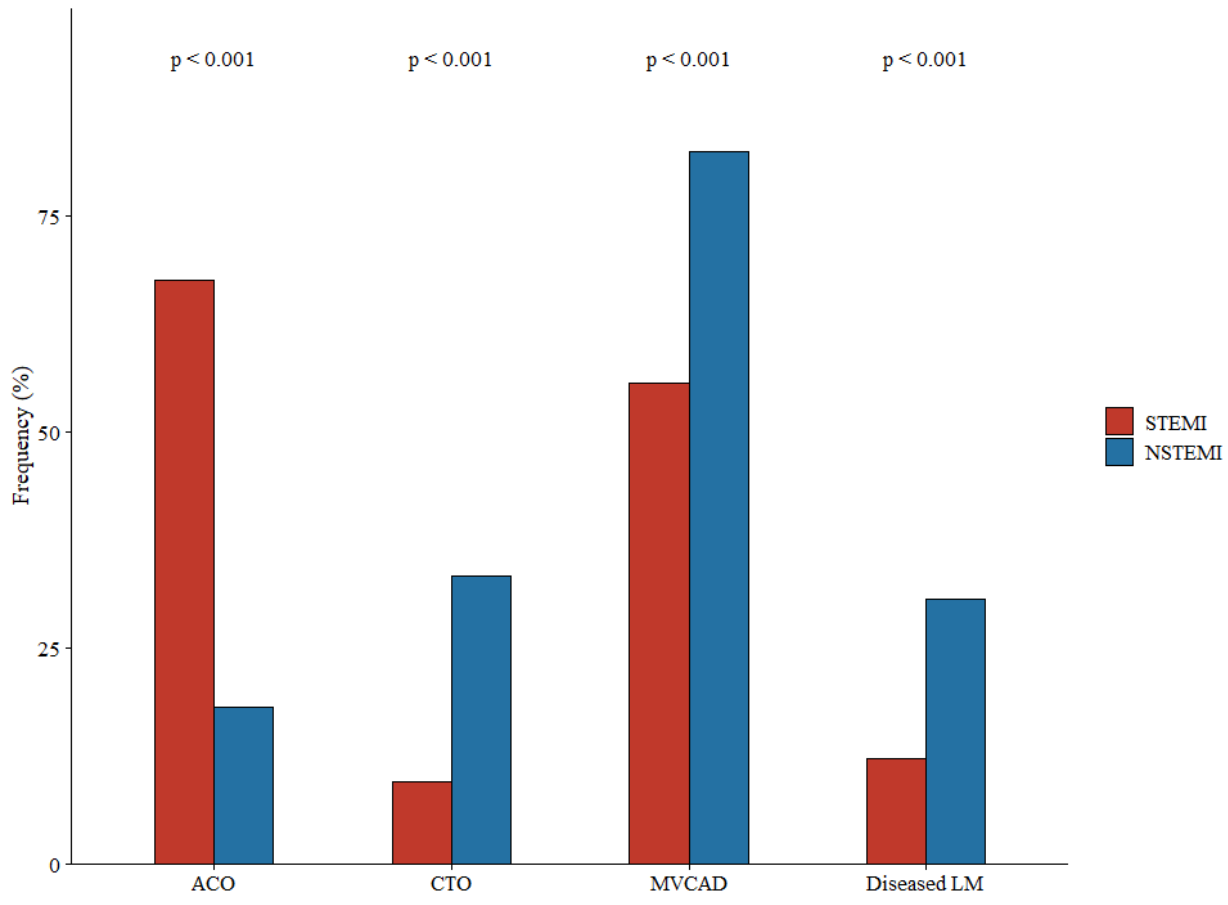


**Supplemental Figure 4. Angiographic characteristics of patients undergoing left heart catheterization**

Bar chart comparing angiographic findings among patients with ST-segment elevation myocardial infarction (STEMI) versus non–ST-segment elevation myocardial infarction (NSTEMI) undergoing left heart catheterization. ACO = acute coronary occlusion; CTO = chronic total occlusion; LM = left main; MVCAD = multivessel coronary artery disease

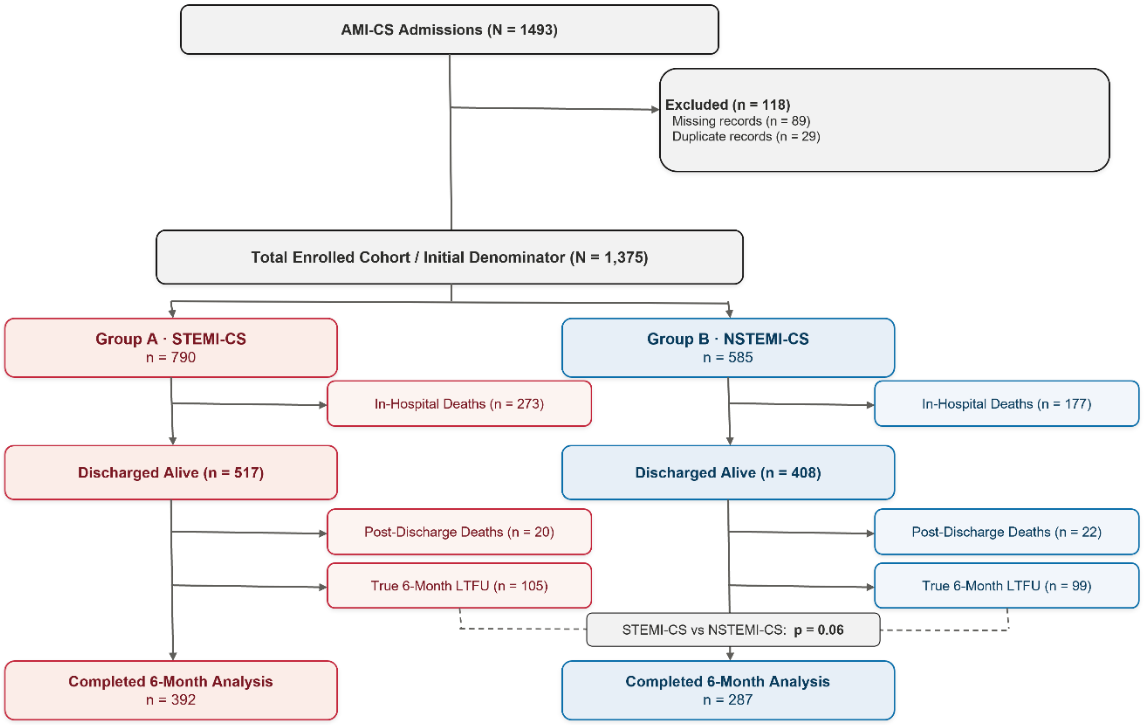


**Supplemental Figure 5. Study flow and 6-month follow-up accounting**

Flowchart depicting distribution of STEMI-CS and NSTEMI-CS. Patients with known vital status totaled 1,171. The Lost to follow-up (LTFU) comparison ($p = 0.06$) tests STEMI-CS vs NSTEMI-CS using the enrolled cohort as the denominator (13.3% vs 16.9%).

**Supplemental Table 1. ICD-10 codes for CS etiologies and management procedures**

| | ICD-10 Diagnosis Codes |
|---|---|
| STEMI | I21.01, I21.02, I21.09, I21.11, I21.19, I21.21, I21.29, I21.3, I22.0, I22.8 |
| NSTEMI | I21.4, I22.2 |

| | |
|---|---|
| Cardiogenic shock | R57.0 |
| LHC | 4A023N7, 4A023N8, B2111ZZ, B211YZZ, B2161ZZ, B216YZZ, B2151ZZ, B215YZZ, B2181ZZ, B218YZZ, B2131ZZ, B213YZZ, B21F1ZZ, B2121ZZ, B2101ZZ, B210110 |
| PAC | 4A023N6, 4A023N8, B2141ZZ, B31S1ZZ, B31U1ZZ, B214YZZ, B2161ZZ, B216YZZ, B2171ZZ, 5A12012, B31TYZZ, 02HR32Z, 02HQ33Z, 02HR33Z, 02HP32Z, 02HQ02Z, 02HQ32Z, 4A133B3, 5A1221Z |
| PCI | 027337Z, 027037Z, 027036Z, 02703FZ, 027035Z, 02703EZ, 0270346, 0270356, 027034Z, 0270456, 02703DZ, 02703Z6, 02703ZZ, 027237Z, 027236Z, 0272356, 0272346, 027234Z, 02723ZZ, 027137Z, 027136Z, 027135Z, 02713EZ, 0271376, 0271366, 0271356, 027134Z, 02713DZ, 02714E6, 02713ZZ, 02C03ZZ, X2C2361, 02C13Z6, X2C1361, 02C13ZZ, 0271346, 0273346, 0270366, 027046Z, 02C00ZZ |
| CABG | 021309W, 02130Z9, 0210093, 0210099, 02100AW, 02100KW, 02100JW, 02100J3, 02100Z3, 02100A9, 02100Z9, 02100Z8, 02100AC, 02100ZC, 0212093, 0212099, 021209W, 0211093, 0211099, 02110AW, 021109W, 021009W, 02110KW, 02110JW, 02100K8, 02110Z9, 02110Z8, 02110AC, 0213099, 02100A8, 021009C, 02120Z9, 02110A8, 02110Z3 |
| IABP | 5A02210, 5A02110 |
| Impella | 5A0221D, 02HA3RZ, 02HA4RZ, 02HA3RJ, 02HA0RZ, 5A0211D, 02HA3RS |
| VA-ECMO | 5A15223, 5A1522G, 5A1522F, 5A02216 |

| | |
|---|---|
| LVAD | 02HA0QZ |
| Mechanical ventilation | 0DH57BZ, 0CHY7BZ, 0BH17EZ, 0BH18EZ, 5A1945Z, 5A1955Z, 5A1935Z, 5A19054, 5A0935Z, 5A09459, 5A0945Z, 5A0955Z |
| Renal replacement therapy | 5A1D00Z, 5A1D60Z, 5A1D70Z, 5A1D80Z, 5A1D90Z |
| Heart Transplant | 02YA0Z0 |
| End-stage renal disease | N18.6, I12.0 |

CS, cardiogenic shock; NSTEMI, non-ST-segment elevation myocardial infarction; STEMI, ST-segment elevation myocardial infarction; LHC, Left Heart Catheterization; PAC, Pulmonary Artery Catheterization; PCI, Percutaneous Coronary Intervention; CABG, Coronary Artery Bypass Grafting; IABP, Intra-Aortic Balloon Pump; VA-ECMO, Veno-Arterial Extracorporeal Membrane Oxygenation; LVAD, Left Ventricular Assist Device; pVAD, percutaneous ventricular assist device

**Supplemental Table 2.** Drop-out analysis. Comparison of baseline characteristics between patients with complete 6-month follow-up and those lost to follow-up.

| | Complete follow-up (N=1171) | LTFU (N=204) | p-value |
|---|---|---|---|
| Age (years) | 69.1 ± 12.8 | 69.1 ± 12.7 | >0.9 |
| Sex: Female | 373 (31.9%) | 59 (28.9%) | 0.405 |
| BMI (kg/m²) | 26.8 (23.9–30.8) | 25.7 (22.8–28.1) | <0.001 |
| AMI-CS type | | | 0.061 |
| NSTEMI | 585 (42.5%) | 486 (41.5%) | |
| STEMI | 790 (57.5%) | 685 (58.5%) | |
| Race | | | 0.180 |
| African American/Black | 112 (9.6%) | 18 (8.8%) | 0.951 |
| Asian | 114 (9.7%) | 22 (10.8%) | 0.368 |
| Other/multiracial | 203 (17.3%) | 42 (20.6%) | 0.078 |
| White | 657 (56.1%) | 90 (44.1%) | 0.043 |
| Transferred from another hospital | 494 (42.2%) | 89 (43.6%) | 0.701 |
| Comorbidities | | | |
| Hypertension | 747 (63.8%) | 126 (61.8%) | 0.579 |
| Chronic kidney disease | 207 (17.7%) | 27 (13.2%) | 0.119 |
| Coronary artery disease | 542 (46.3%) | 76 (37.3%) | 0.017 |
| Type 2 diabetes mellitus | 470 (40.1%) | 89 (43.6%) | 0.349 |
| Atrial fibrillation | 265 (22.6%) | 43 (21.1%) | 0.624 |

| | | | |
|---|---|---|---|
| CCI total score | 5.5 ± 3.2 | 5.6 ± 3.1 | 0.815 |
| LVEF, initial (%) | 30.0 (20.0–40.0) | 30.0 (23.0–43.0) | 0.412 |
| APACHE score, initial | 85.4 ± 37.9 | 79.5 ± 34.0 | 0.072 |
| Cardiac arrest on admission | 65 (5.6%) | 4 (2.0%) | 0.030 |
| SCAI stage on admission | | | <0.001 |
| A | 169 (14.4%) | 34 (16.7%) | 0.330 |
| B | 172 (14.7%) | 39 (19.1%) | 0.076 |
| C | 157 (13.4%) | 32 (15.7%) | 0.312 |
| D | 289 (24.7%) | 59 (28.9%) | 0.136 |
| E | 354 (30.2%) | 30 (14.7%) | <0.001 |
| MCS | 754 (64.4%) | 131 (64.2%) | 0.962 |
| LHC | 968 (82.7%) | 175 (85.8%) | 0.272 |
| PCI* | 554 (57.2%) | 106 (60.6%) | 0.410 |
| CABG | 213 (18.2%) | 41 (20.1%) | 0.517 |

Complete follow-up denotes patients with ascertained 6-month vital status via encounter availability on the Northwell health records; Lost to follow-up (LTFU) denotes those without documentation. Comparisons suggest that LTFU was random.

Continuous variables presented as mean ± SD or median (Q1, Q3); categorical variables as n (%). P-values reflect Student's t-test, Mann-Whitney U, or chi-square / Fisher's exact, as appropriate.

*Percutaneous coronary intervention is expressed as a percentage of patients who underwent left heart catheterization (Confirmed n=968, LTFU n=175); all other percentages use the full group total.

AMI-CS = Acute Myocardial Infarction Cardiogenic Shock, APACHE = Acute Physiology and Chronic Health Evaluation score, BMI = Body Mass Index, CABG = Coronary Artery Bypass Grafting, CCI = Charlson Comorbidity Index, LHC = Left Heart Catheterization, LTFU = Lost to Follow-Up, LVEF = Left Ventricle Ejection Fraction, MCS = Mechanical Circulatory Support, NSTEMI = non-ST-segment Elevation Myocardial Infarction, PCI = Percutaneous Coronary Intervention, SCAI = Society for Cardiovascular Angiography and Interventions, STEMI = ST-segment Elevation Myocardial Infarction
